# Cartilage oligomeric matrix protein as a marker of progressive liver fibrosis in biliary atresia

**DOI:** 10.1038/s41598-021-95805-x

**Published:** 2021-08-17

**Authors:** Wanvisa Udomsinprasert, Napat Angkathunyakul, Jiraphun Jittikoon, Usa Chaikledkaew, Paisarn Vejchapipat, Yong Poovorawan, Sittisak Honsawek

**Affiliations:** 1 Department of Biochemistry, Faculty of Pharmacy, Mahidol University, 447 Sri-Ayudthaya Road, Rajathevi, Bangkok, 10400 Thailand; 2 Department of Pathology, Faculty of Medicine Siriraj Hospital, Mahidol University, Bangkok, 10700 Thailand; 3 Social and Administrative Pharmacy Division, Department of Pharmacy, Faculty of Pharmacy, Mahidol University, Bangkok, 10400 Thailand; 4 Mahidol University Health Technology Assessment (MUHTA) Graduate Program, Mahidol University, Bangkok, 10400 Thailand; 5 Department of Surgery, Faculty of Medicine, King Chulalongkorn Memorial Hospital, Thai Red Cross Society, Chulalongkorn University, Bangkok, 10330 Thailand; 6 Department of Pediatrics, Faculty of Medicine, Center of Excellence in Clinical Virology, King Chulalongkorn Memorial Hospital, Chulalongkorn University, Bangkok, 10330 Thailand; 7 Osteoarthritis and Musculoskeleton Research Unit, Department of Biochemistry, Faculty of Medicine, King Chulalongkorn Memorial Hospital, Thai Red Cross Society, Chulalongkorn University, Bangkok, 10330 Thailand

**Keywords:** Biomarkers, Diseases, Medical research

## Abstract

This study aimed to determine whether mRNA and protein levels of cartilage oligomeric matrix protein (COMP), a glycoprotein responsible for modulating homeostasis of extracellular matrix, in the systemic and local liver environments were associated with clinical parameters of biliary atresia (BA) patients and might serve as a biomarker for BA severity. COMP protein levels in the circulation of 96 BA patients and 56 healthy controls and its mRNA and protein expressions in the liver of 20 BA patients and 5 non-BA patients were evaluated using enzyme-linked immunosorbent assay, real-time polymerase chain reaction, and immunohistochemistry, respectively. In the circulation of BA patients, COMP levels were significantly higher than those in healthy controls. Compared with early-stage BA patients, those with advanced-stage including jaundice, fibrosis, and hepatic dysfunction had significantly increased circulating COMP levels. Raised circulating COMP levels were found to be independently correlated with degree of liver fibrosis. Survival analysis showed that elevated circulating COMP levels were significantly associated with decreased survival of BA patients. Receiver-operating characteristic curve analysis unveiled a diagnostic value of circulating COMP as a non-invasive biomarker of BA (AUC = 0.99), with a sensitivity of 100.0% and a specificity of 98.2%. In the liver, both COMP mRNA and protein expressions of BA patients with fibrosis were significantly greater than those of BA patients without fibrosis and non-BA patients. Collectively, increased circulating COMP might reflect unfavorable outcome of BA patients and have potential as a novel biomarker for the disease severity following Kasai-operation.

## Introduction

Biliary atresia (BA) is a severe hepatobiliary disease of neonate pathologically characterized by devastating fibroinflammatory destruction of the extra- and the intra-hepatic bile ducts, leading to severe cholestasis, cirrhosis, and eventually to hepatic failure. Despite being a rare disease, BA is the most common indicator for pediatric liver transplantation^1^. In an attempt to restore bile flow, Kasai hepato-portoenterostomy (KPE) is traditionally performed, which reportedly increases survival rate with native liver and improves long-term clinical outcomes^2^. However, owing to delays in definitive diagnosis and lack of non-invasive biomarkers, most patients develop persistent fibrosis and progress to end-stage liver disease, which require liver transplantation for prolonged survival^3^. For that reason, assessment of progressive liver fibrosis is essential for the management of post-operative BA patients. It is important to note that non-invasive diagnostic indicators for progressive fibrosis may facilitate the differential diagnosis and evaluation of postoperative prognosis, which may be helpful in improving clinical outcomes of BA patients following KPE and delaying or even avoiding need for liver transplantation.

Given that a key event participating in liver fibrogenesis is accumulation of extracellular matrix (ECM) components, cartilage oligomeric matrix protein (COMP), a non-collagenous extracellular matrix protein, is gaining increasing interest as a possible mediator for liver fibrosis. As a calcium-binding glycoprotein mainly found in the ECM of skeletal tissue^4^, COMP exerts an important role in modulating the cellular phenotype during tissue genesis and remodeling^5–7^, thus highlighting its possible action in liver fibrogenesis. Regarding this, COMP has been reported to enhance collagen-1 deposition in hepatic stellate cells (HSCs) via CD36 receptor signaling and activation of mitogen-activated protein kinase (MEK)1/2-phosphorylated extracellular signal-related kinase (pERK)1/2 pathway, thereby establishing pro-fibrogenic effect of COMP^8^. More precisely, increased COMP expression has been reportedly associated with fibrogenesis in a wide range of tissues—especially in the liver^9–11^. In addition to this, it has been shown that circulating COMP levels were positively correlated with degree of liver fibrosis in patients with chronic hepatitis C virus (HCV) infection^12^. Altogether, the aforementioned results lend further support to the view that circulating COMP may have a potential as a non-invasive biomarker for progressive liver fibrosis in post-operative BA patients.

Although an involvement of COMP in pathogenesis of liver fibrosis has been clarified, whether its protein levels in the systemic and local environments were associated with severity of liver fibrosis in post-operative BA patients remains to be determined. Accordingly, the purpose of this study was to measure circulating CLU levels in post-operative BA patients compared to age-matched healthy controls and to determine the possible application of circulating COMP as a non-invasive marker of liver fibrosis in those patients. Furthermore, we investigated protein expression and localization of COMP in BA livers with and without the fibrotic scarring.

## Materials and methods

The study protocol conducted in accordance with the ethical standards outlined in the Declaration of Helsinki was approved by the Institutional Review Board of the Faculty of Medicine, Chulalongkorn University and the Faculty of Dentistry/Faculty of Pharmacy, Mahidol University. Written informed consent was acquired from the participants’ guardian.

### Study participants

A total of 96 post-operative BA patients and 56 age-matched unaffected volunteers were recruited in this retrospective case–control study, as previously detailed^13^. All BA patients were diagnosed by intraoperative cholangiography and were surgically treated with original Kasai operation. Healthy controls who attended the Well Baby Clinic at King Chulalongkorn Memorial Hospital for vaccination had normal physical findings and no underlying disease. In terms of bile flow establishment, BA patients were stratified with regards to serum total bilirubin (TB) into non-jaundice (TB < 2 mg/dL, *n* = 61) and persistent jaundice groups (TB ≥ 2 mg/dL, *n* = 35). In the context of severity of liver fibrosis (liver stiffness values), the patients were also classified into no fibrosis (< 7.1 kPa, *n* = 20) and fibrosis groups (≥ 7.1 kPa, *n* = 76). According to alanine aminotransferase (ALT) values indicating severity of hepatic injury, BA patients were divided into early-stage (ALT < 100 IU/L, *n* = 46) and late-stage groups (ALT ≥ 100 IU/L, *n* = 50). Regarding their jaundice and fibrosis statuses, the patients were categorized into favorable outcome (TB < 2 mg/dL, < 7.1 kPa, *n* = 18) and unfavorable outcome groups (TB ≥ 2 mg/dL, ≥ 7.1 kPa, *n* = 33), based on their values of TB and liver stiffness.

### Clinical assessments of outcomes

After overnight fast, peripheral venous blood samples were drawn from all participants into ethylenediaminetetraacetic acid and clot blood tubes for routine laboratory tests including aspartate aminotransferase (AST), ALT, alkaline phosphatase (ALP), serum albumin, TB, direct bilirubin (DB), and albumin. All the aforementioned tests were performed on a Roche Hitachi 912 chemistry analyzer (Roche Diagnostics, Basel, Switzerland). Measurement of liver stiffness by transient elastography was performed using a Fibroscan (Echosens, Paris, France), as previously delineated^13^. Briefly, the assessments were performed by placing a Fibroscan transducer probe on the intercostal space at the area of the right lobe of the liver with patients lying in a dorsal decubitus position with maximum abduction of the right arm. Measurements were then conducted until 10 validated results were obtained with a success rate of at least 80%. The median value of 10 validated scores represented the elastic modulus measurement of the liver, which was expressed in kilopascals (kPa).

### Quantitation of circulating COMP levels

Circulating COMP levels were quantified using a commercial sandwich enzyme-linked immunosorbent assay (ELISA) kit (R&D Systems, Minneapolis, MN, United States). According to manufacturer’s instructions previously described^14^, recombinant human COMP standards, plasma samples were pipetted into every well of a microplate, which was precoated with specific antibody to COMP. After incubating for 2 h. at room temperature, all wells were washed completely 3 times with washing reagent. Sequentially, COMP conjugate was pipetted into every well and incubated for 2 h. at room temperature. After 3 washes, substrate solution was added into each well, and then the microplate was incubated for 20 min at room temperature without light. Finally, all reactions were terminated by the stop reagent, and the optical density was evaluated using automated microplate reader at 450 nm. The intensity of color derived is clearly proportionate to the quantity of COMP in the samples. For the technical validity of measurements, plasma samples were analyzed for human COMP at a 1000-fold dilution.

### Assessment of *COMP* mRNA expression

Perioperative liver biopsies of 20 BA patients who underwent KPE and 5 non-BA patients who suffered from choledochal cysts and underwent liver biopsies with no signs of fibrosis were harvested at the Department of Surgery, King Chulalongkorn Memorial Hospital. Total RNA was extracted from liver biopsies using a RNeasy Mini Kit (Qiagen, Hilden, Germany) with cDNA reverse transcribed using TaqMan Reverse Transcription Reagents (Applied Biosystems, Inc., Foster City, CA, USA). Real-time polymerase chain reaction (PCR) was performed using SYBR Green fluorescence (biotechrabbit GmbH, Hennigsdorf, Germany) on a StepOnePlus Real-Time PCR System (Applied Biosystems, Inc., Foster City, CA, USA). The primers used for *COMP* and glyceraldehyde 3-phosphate dehydrogenase (*GAPDH*) amplifications were, as follows: forward *COMP*, 5′-TGG-GTT-ACT-GCC-TTC-AAT-G-3′; reverse *COMP*, 5′-GTT-GTG-TCC-AAG-ACC-ACG-TT-3′; forward *GAPDH*, 5′-GTG-AAG-GTC-GGA-GTC-AAC-GG-3′; reverse *GAPDH*, 5′-TCA-ATG-AAG-GGG-TCA-TTG-ATG-G-3′. Relative *COMP* mRNA expressions were normalized to *GAPDH* as an internal control and were determined using the 2^−∆∆Ct^ method.

### Determination of COMP protein expression

Localization of COMP protein expression in BA livers was determined using immunohistochemical analysis. Liver specimens were paraffin-embedded and subsequently sectioned, according to standard protocols previously determined^15^. Routine staining with hematoxylin and eosin (H&E) and immunohistochemistry staining with antibodies was performed to detect COMP protein expression (Abcam, Cambridge, MA, USA). A standard immunohistochemical technique was performed using a Ventana Benchmark XT autostainer (Ventana Medical Systems Inc., Tucson, AZ, USA). Briefly, tissue sections were deparaffinized and rehydrated. Endogenous peroxidase activity was blocked by 0.3% hydrogen peroxide for 10 min. Following heat-induced antigen retrieval in 10 mmol/L citrate buffer (pH 6.0) for 5 min, the slides were incubated in pepsin for 7 min and subsequently incubated with 1:500 diluted primary antibodies for 2 h. Afterwards, the sections were stained with the secondary antibody conjugated to streptavidin/horseradish peroxidase for 45 min at room temperature. Reaction products were visualized using 3,3-diaminobenzidine tetrahydrochloride (Sigma, St. Louis, MO, USA), and the sections were counterstained with hematoxylin.

### Masson’s trichrome staining

To determine collagen fibers indicating progression of liver fibrosis in the liver biopsies of BA patients, Masson’s Trichrome staining was conducted according to the manufacturer’s protocol (Genmed Scientifics, Wilmington, DE). Collagen fibers were stained blue, nuclei were stained black, and the background was stained red. Degree of liver fibrosis was evaluated according to the Metavir grading system^16^, as follows: F0, no fibrosis; F1, mild fibrosis in the portal area; F2, mild bridging fibrosis in the adjacent portal area; F3, severe bridging fibrosis in the adjacent portal area; and F4, cirrhosis and annular fibrosis with nodule formation.

### Statistical analysis

All statistical analyses were executed using SPSS Statistics version 22.0 (SPSS Inc., Chicago, IL, USA). Comparisons in means were assessed by Student’s *t*-test (for 2 groups) and one-way analysis of variance (ANOVA, > 2 groups) with a Tukey post hoc test, while comparisons in abnormally distributed continuous variables were accomplished using Mann–Whitney *U* test and Kruskal–Wallis *H* test where appropriate. Correlations between circulating COMP levels and clinical parameters were evaluated using Spearman’s rho correlation coefficient (*r*). To exclude or control confounding variables that can interfere the outcome, multivariate logistic regression models were performed. Kaplan–Meier analysis with end points of death was undertaken to estimate the survival function, in which the differences in survival curves were determined using Log-rank test. Receiver operating characteristic (ROC) curve was constructed for the estimation of the area under the ROC curve (AUC), sensitivity, and specificity, indicating the feasibility of using circulating COMP as a possible biomarker for post-operative BA. Data are presented as mean ± standard deviation (SD). For differences and correlations, a *P*-value less than 0.05 (based on a two-tailed test) was considered statistically significant.

## Results

### Baseline and clinical characteristics of study subjects

Baseline demographic and clinical characteristics of 96 post-Kasai BA patients and 56 healthy volunteers are summarized in Table 1. Both groups were matched for age and sex. As expected, BA patients had significantly greater values of clinical parameters including liver stiffness, AST, and ALT than healthy controls (*P* < 0.001).

**Table 1:** Baseline and clinical characteristics of study participants.

Variables	Healthy controls (*n* = 56)	BA patients (*n* = 96)	*P*-value
Age (years)	8.92 ± 0.47	10.00 ± 6.17	0.59
Gender (male: female)	32:24	50:46	0.55
BMI (kg/m^2^)	15.78 ± 2.19	17.23 ± 3.07	0.53
Liver stiffness (kPa)	4.05 ± 0.11	31.02 ± 2.38	< 0.001*
AST (IU/L)	27.12 ± 0.89	133.72 ± 9.90	< 0.001*
ALT (IU/L)	9.16 ± 0.75	127.04 ± 9.12	< 0.001*
ALP (IU/L)	–	425.85 ± 34.05	NA
Albumin (g/dL)	–	4.07 ± 0.84	NA
TB (mg/dL)	–	2.01 ± 1.41	NA
DB (mg/dL)	–	1.70 ± 1.32	NA

*ALP* alkaline phosphatase, *ALT* alanine aminotransferase, *APRI* AST to platelet ratio index, *AST* aspartate aminotransferase, *BA* biliary atresia, *BMI* body mass index, *DB* direct bilirubin, *TB* total bilirubin, *NA* not available.

*Difference is considered statistically significant at *P*-value less than 0.05 (two-tailed).

### Increased circulating COMP levels in BA subjects and those with advanced-stage

**Figure 1:**
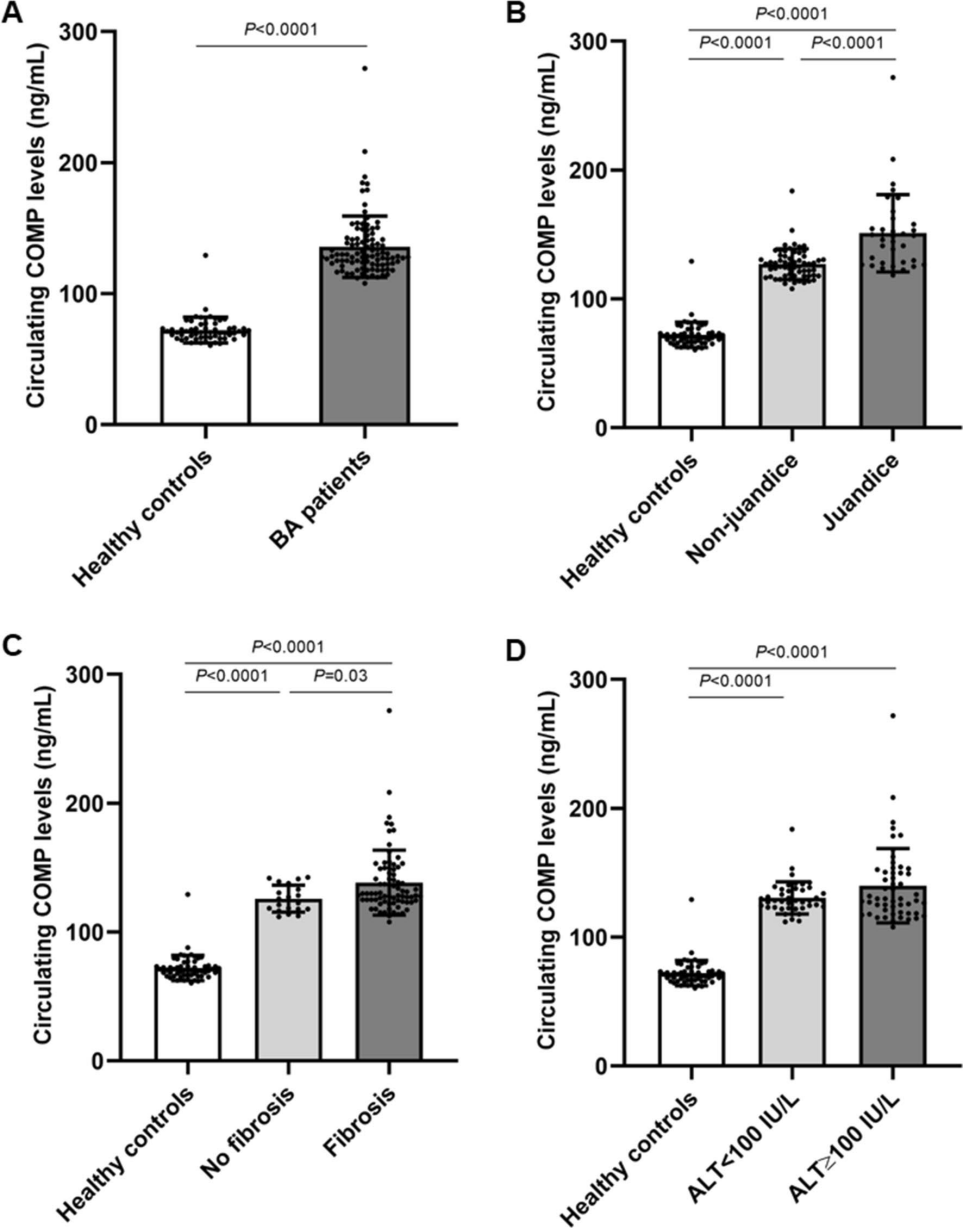
Circulating COMP levels in healthy controls and BA patients with different groups. (**A**) Healthy controls (*n* = 56) and BA patients (*n* = 96). (**B**) BA patients divided into non-jaundice (TB < 2 mg/dL, *n* = 61) and jaundice (TB ≥ 2 mg/dL, *n* = 35) groups. (**C**) BA patients classified into no fibrosis (< 7.1 kPa, *n* = 20) and fibrosis (≥ 7.1 kPa, *n* = 76) groups. (**D**) BA patients categorized into low- (< 100 IU/L, *n* = 46) and high ALT values (≥ 100 IU/L, *n* = 50).

As depicted in Fig. 1A, circulating COMP levels were significantly higher in BA patients than those in healthy controls (*P* < 0.001). In analyses stratified by BA severity, the patients with advanced-stage including jaundice, fibrosis, and high ALT values exhibited remarkably more pronounced COMP levels in the circulation (Fig. 1B–D). In regards to jaundice status, BA patients with jaundice showed significantly augmented circulating COMP levels when compared with those with jaundice-free and healthy volunteers (*P* < 0.001, *P* < 0.001, respectively) (Fig. 1B). In conformity with this, circulating COMP levels were significantly greater in BA patients with non-jaundice than those in healthy controls (*P* < 0.001) (Fig. 1B). When severity of liver fibrosis was considered, circulating COMP levels were significantly elevated in BA patients with fibrosis, compared with the patients without fibrosis and healthy controls (*P* = 0.03, *P* < 0.001, respectively) (Fig. 1C). Correspondingly, circulating COMP levels remained significantly increased in BA patients without liver fibrosis, as compared to healthy controls (*P* < 0.001) (Fig. 1C). In stratified analysis by ALT values reflecting severity of hepatocellular damage, despite higher circulating COMP levels in BA patients with high ALT levels (≥ 100 IU/L) than those in the patients with low ALT levels (< 100 IU/L), this difference was not statistically significant (Fig. 1D). Compared to healthy controls, a remarkable increment in circulating COMP levels was found in both BA patients with high and low ALT levels (*P* < 0.001, *P* < 0.001, respectively) (Fig. 1D).

### Significant associations between circulating COMP levels and clinical parameters

Relationships between circulating COMP levels and clinical parameters in BA patients are detailed in Table 2. Spearman’s rho correlation analysis demonstrated that circulating COMP levels were positively correlated with liver stiffness (*r* = 0.54, *P* < 0.001), AST (*r* = 0.45, *P* < 0.001), ALT (*r* = 0.35, *P* < 0.001), ALP (*r* = 0.32, *P* < 0.001), TB (*r* = 0.44, *P* < 0.001), and DB (*r* = 0.41, *P* < 0.001) in BA patients. On the other hand, circulating COMP levels were found to be inversely associated with albumin in BA patients (*r* =  − 0.23, *P* = 0.047).

**Table 2:** Spearman’s rho correlation and multivariate linear regression analyses determining associations between circulating COMP levels and clinical parameters in BA patients.

Variables	Circulating COMP levels (ng/mL)			
	Spearman’s rho correlation		Linear regression^a^	
	Coefficient (*r*)	*P*-value	β coefficient (95% CI)	*P*-value
Age (years)	− 0.20	0.08	**–**	**–**
BMI (kg/m^2^)	0.12	0.38	**–**	**–**
Liver stiffness (kPa)	0.54	< 0.001*	0.33 (0.13 to 0.52)	0.002*
AST (IU/L)	0.47	< 0.001*	**–**	**–**
ALT (IU/L)	0.35	< 0.001*	**–**	**–**
ALP (IU/L)	0.32	0.002*	**–**	**–**
Albumin (g/dL)	− 0.23	0.047*	**–**	**–**
TB (mg/dL)	0.44	< 0.001*	**–**	**–**
DB (mg/dL)	0.41	< 0.001*	**–**	**–**

*ALP* alkaline phosphatase, *ALT* alanine aminotransferase, *APRI* AST to platelet ratio index, *AST* aspartate aminotransferase, *BA* biliary atresia, *BMI* body mass index, *DB* direct bilirubin, *TB* total bilirubin, *NA* not available.

*Correlation is considered statistically significant at *P*-value less than 0.05 (two-tailed).

^a^The coefficient was adjusted for age, BMI, AST, ALT, ALP, albumin, TB, and DB.

To subsequently attest independent associations between circulating COMP levels and outcome variables in BA patients, multivariate linear regression analysis was undertaken. After adjustments for confounding factors including age, gender, BMI, and clinical variables consisting of AST, ALT, ALP, TB, DB, and albumin, an elevation in circulating COMP levels was shown to be independently related to increased values of liver stiffness (β-coefficient = 0.33; 95% CI 0.13 to 0.52; *P* = 0.002) (Table 2).

### Decreased survival in BA patients with unfavorable outcome along with high circulating COMP levels

**Figure 2:**
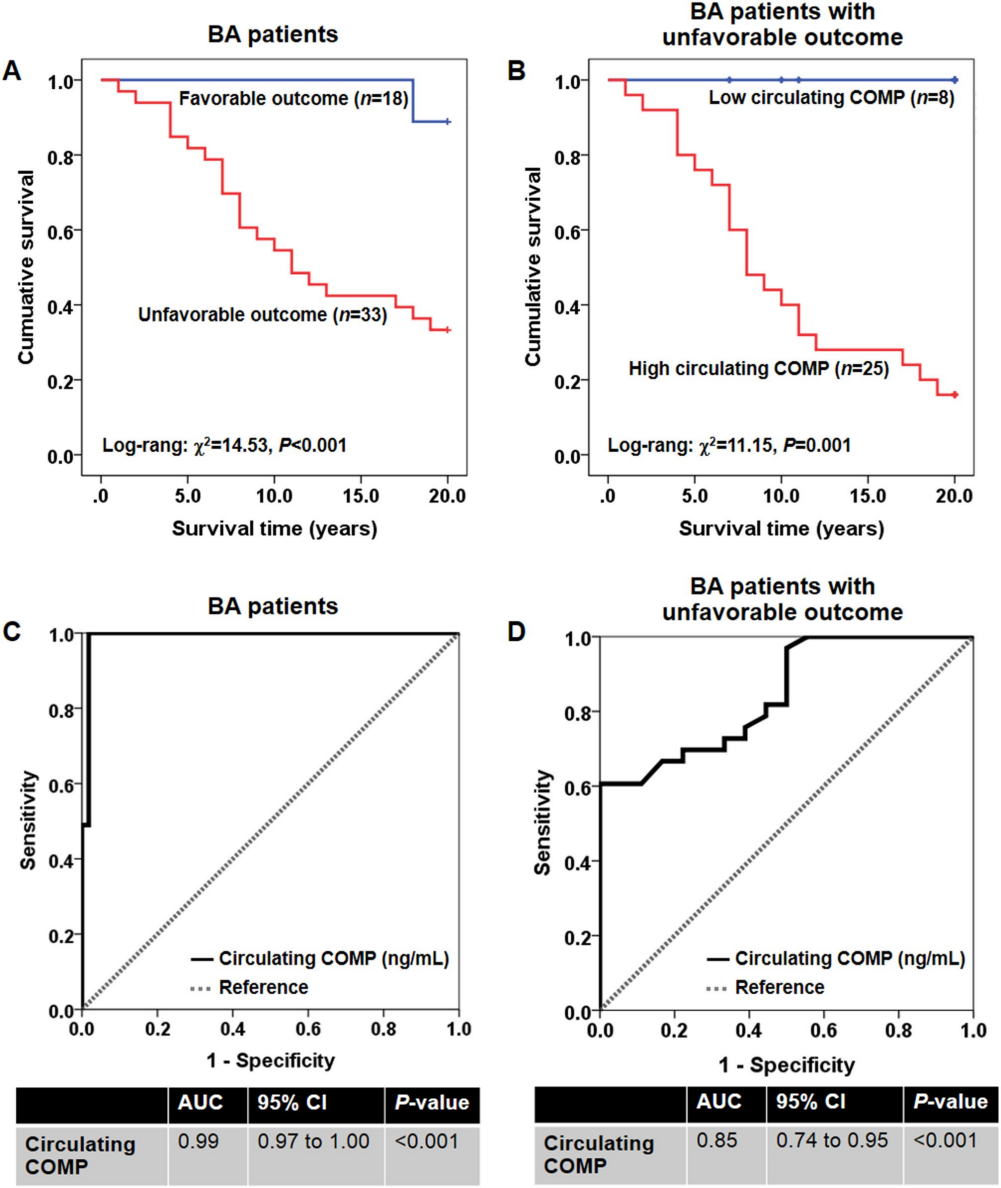
Kaplan–Meier survival curve of post-operative BA patients and receiver operating characteristic curve demonstrating diagnostic value of circulating COMP in BA patients. (**A**) Comparing survival curves between BA patients with favorable and unfavorable outcomes. (**B**) Comparing survival curves between low and high circulating COMP levels in BA patients with unfavorable outcome. (**C**) Possibility of circulating COMP as a biomarker for distinguishing BA patients from healthy controls. (**D**) Possibility of circulating COMP as a biomarker for discriminating BA patients with unfavorable outcome from favorable outcome following KPE.

Given a significant increase in circulating CLU levels in BA patients—especially in those with poor outcome, Kaplan–Meier analysis was performed to evaluate the possible effect of higher circulating CLU levels on reduced survival rate in BA patients with unfavorable outcome. In survival analysis stratified by their clinical outcome following KPE with regards to jaundice and fibrosis statuses, Kaplan–Meier analysis showed that the 20-year survival rate of BA patients with unfavorable outcome (66.7%) was significantly lower than that of the patients with favorable outcome (11.1%) (Log-rank: χ^2^ = 14.53, *P* < 0.001) (Fig. 2A). When divided into low and high circulating COMP levels based on its median distribution in BA patients with the cut-off value of 128.47 ng/mL, a significant reduction in survival rate was detected in BA patients with unfavorable outcome combined with high circulating COMP levels (84.0%), compared with those with low circulating COMP levels (0%) (Log-rank: χ^2^ = 11.2, *P* = 0.001) (Fig. 2B).

### Circulating COMP as a possible biomarker for developmental and progressive BA

Whether circulating COMP could be used as a non-invasive biomarker for BA development was determined using the ROC curve analysis. As shown in Fig. 2C, the analysis displayed that the optimal cutoff value of circulating COMP as a useful biomarker for discriminating BA patients from healthy control was projected to be 97.80 ng/mL, which provided a sensitivity of 100.0%, a specificity of 98.2%, and an AUC of 0.99 (95% CI 0.97 to 1.00, *P* < 0.001).

The possibility whether circulating COMP levels allowed differentiating BA patients with unfavorable outcome from those with favorable outcome was further assessed. Based on the ROC curve represented in Fig. 2D, the optimal cutoff value of circulating COMP was defined at 143.47 ng/mL, and the AUC was 84.5 (95% CI 0.74 to 0.95; *P* < 0.001). Sensitivity and specificity of circulating COMP as a biomarker for monitoring unfavorable outcome of BA patients following KPE were 60.6% and 100.0%, respectively.

### Up-regulation of COMP mRNA and protein expressions in BA livers with fibrotic scarring

**Figure 3:**
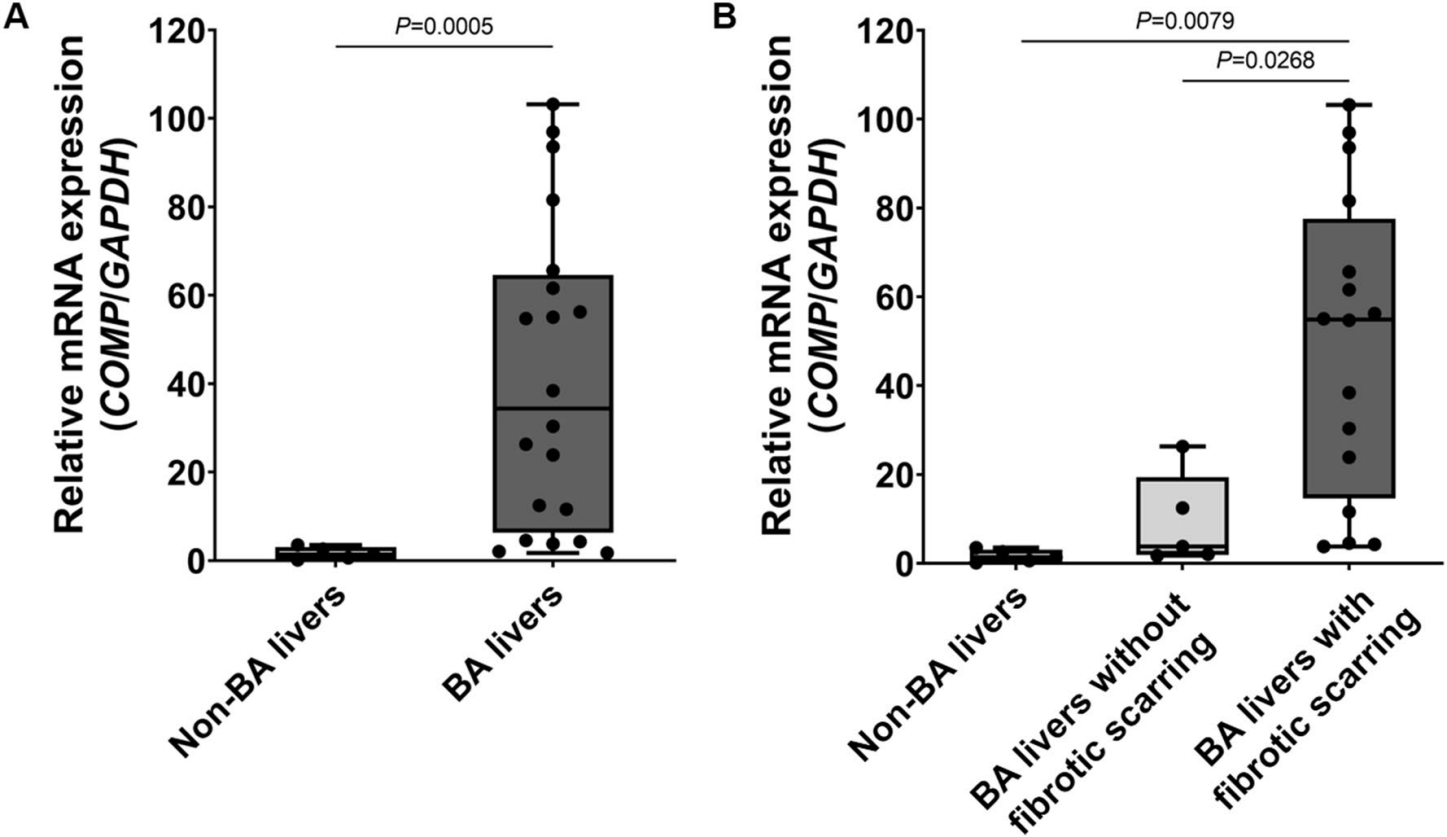
Relative *COMP* mRNA expression in non-BA livers and BA livers with and without the fibrotic scarring. (**A**) Non-BA livers (*n* = 5) and BA livers (*n* = 20). (**B**) BA livers without fibrotic scarring (*n* = 5) and with fibrotic scarring (*n* = 15).

Due to an increment in COMP protein levels in the circulation of BA patients, particularly those with unfavorable outcome, its mRNA and protein expressions in the liver biopsies of BA patients were additionally determined. Out of 96 BA patients, 20 were included in the liver biopsy cohort. Of these, 15/20 (75%) had a Metavir score ≥ 1 (F1–4) indicating fibrosis. Relative *COMP* mRNA expression is depicted in Fig. 3A,B. Compared to non-BA livers, relative *COMP* mRNA expression was significantly up-regulated in BA livers (*P* = 0.008) (Fig. 3A). In stratified analysis by fibrosis status, BA patients with fibrosis exhibited significantly higher relative *COMP* mRNA expression in the liver than those without fibrosis (*P* = 0.027) (Fig. 3B).

**Figure 4:**
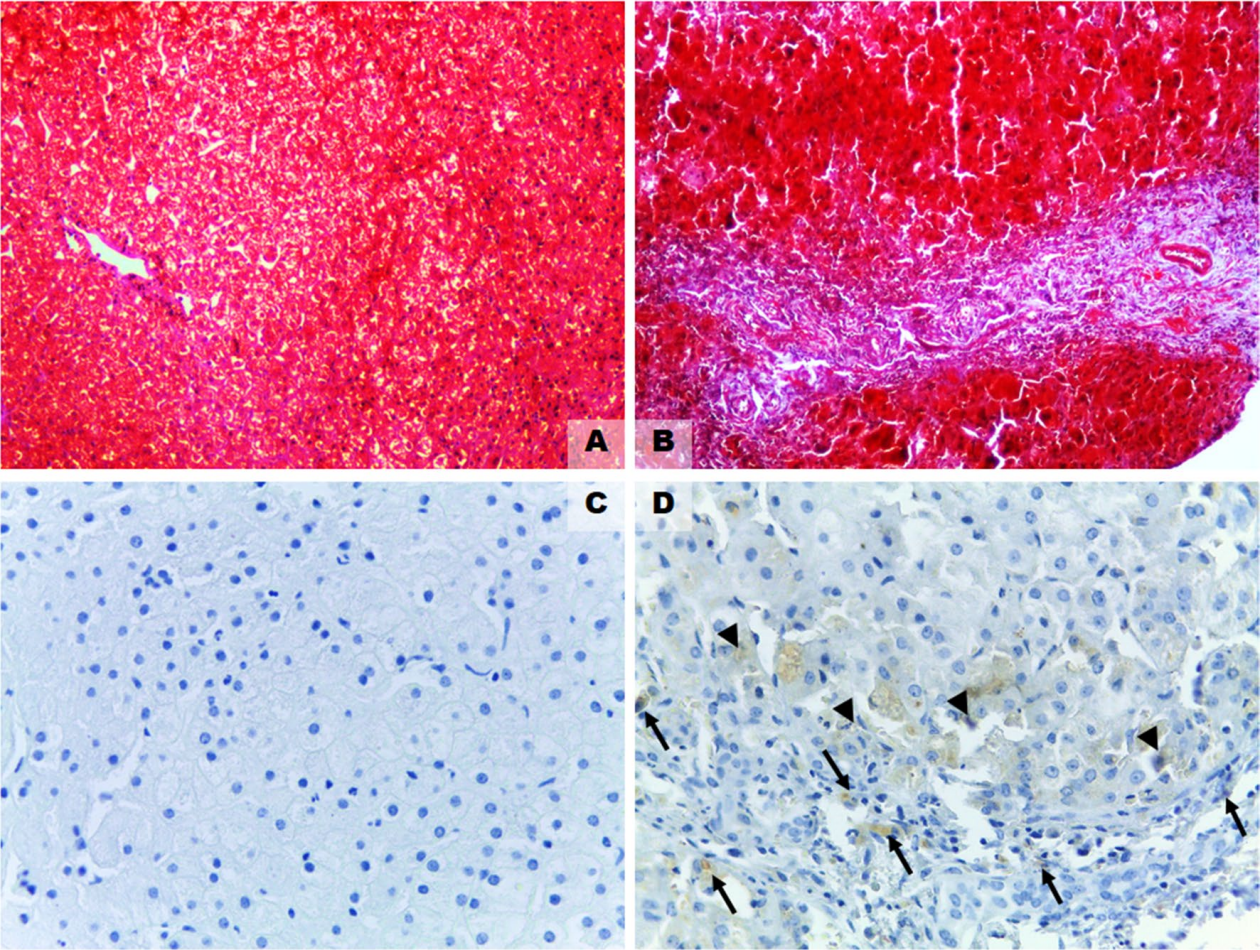
Histological staining with Masson’s Trichrome and immunohistochemistry in BA livers with and without the fibrotic scarring (original magnifications × 400). (**A**) Masson’s Trichrome staining in BA livers without fibrotic scarring. (**B**) Masson’s Trichrome staining in BA livers with fibrotic scarring. (**C**) Immunohistochemical staining for COMP protein expression in BA livers without fibrotic scarring. (**D**) Immunohistochemical staining for COMP protein expression in BA livers with fibrotic scarring.

Histological staining with Masson’s Trichrome and immunohistochemistry is revealed in Fig. 4A–D. Out of 20 BA liver biopsies, 15 those with fibrotic scarring were immunoreactive for COMP. In contrast to this, COMP protein expression was undetectable in the livers of BA patients without liver fibrosis (Metavir score < 1, F0), as unveiled in Fig. 4C. In most COMP positive cases, COMP protein expression was detectable in the periportal hepatocytes (arrows) and the portal macrophages (arrowheads) adjacent to the fibrotic liver area, as illustrated in Fig. 4D.

## Discussion

Progressive liver fibrosis has been well-recognized as the most important predictor of poor outcome of BA patients following KPE. Given a lack of validated non-invasive tools for monitoring liver fibrosis, most BA patients undergo liver transplantation for long-term survival. For this reason, it seems likely that early diagnosis and non-invasive detection of liver fibrosis may pave the way for improved long-term survival after the Kasai surgery. It is necessary to identify close links between alternative molecules known to be implicated in fibrogenesis, liver fibrosis assessed by transient elastography, and other clinical as well as routinely performed biochemical parameters, which may open the door to discovery of specific biomarkers for liver fibrogenesis. From this, in the present study, we investigated whether a component of ECM namely COMP, which has been demonstrated to be significantly increased in the circulation of BA patients, especially those with fibrosis, may have a potential as a non-invasive biomarker of liver fibrosis. Notably, we also found that elevated circulating COMP levels were independently associated with degree of liver fibrosis in BA patients. Our findings have been supported by a previous clinical study revealing a positive association between serum COMP concentrations and stage of liver fibrosis in HCV-infected patients^12^. Alongside this, it has been reported that COMP expression was detectable in a wide spectrum of fibrous connective tissues including the liver^9–11,17^. Consistent with these previous findings, our further result derived from immunohistochemistry displayed the presence of COMP protein expression in BA livers, predominantly in the fibrotic areas. Aside from its protein expression in the livers, *COMP* mRNA expression in BA livers with fibrotic scarring was detected to be significantly greater than that in those without fibrotic scarring and non-BA livers. All above-mentioned findings shed light on not only a significant involvement of COMP in BA pathology including liver fibrosis, but also its potential as a specific biomarker for liver fibrosis in post-operative BA patients. In support of this hypothesis, we further explored whether circulating COMP may be utilized as a non-invasive biomarker for BA patients’ unfavorable outcome with regards to jaundice and fibrosis statues. The ROC curve analysis unveiled that circulating COMP could be employed as a useful biomarker for monitoring unfavorable outcome of BA patients following KPE, which has been attested by a recent study conducted by Zachou et al.^18^ denoting serum COMP as a novel non-invasive biomarker for liver fibrosis in patients with HCV infection. In parallel with this finding, our additional analysis showed that high circulating COMP levels were significantly associated with decreased survival of BA patients with unfavorable outcome. These results support the notion that circulating COMP may have the potential as a novel biomarker for monitoring BA progression—especially liver fibrosis.

In the light of the foregoing considerations, it is tempting to postulate that an elevation in circulating COMP levels in BA patients and those with advanced-stage might reflect compensatory mechanisms of the body that become activated in response to an imbalance in dynamic remodeling of ECM contributing to liver fibrosis. This speculated fate may lead to up-regulated mRNA and protein expressions of COMP in the fibrotic liver tissues, in addition to increased circulating COMP levels in BA patients including those with fibrosis. On the basis of its primary action as a glycoprotein mainly found in the ECM, COMP reportedly not only up-regulated expression of matrix metalloproteinases, key enzymes regulating ECM degradation, but also increased collagen-I deposition in HSCs via CD36 receptor-activated the MEK1/2-pERK1/2 pathway, thereby establishing pro-fibrogenic effect of COMP in the liver^8^. This circumstance may help us explain why increased COMP protein levels were found to be related to degree of liver fibrosis in BA patients. However, the molecular mechanisms underpinning COMP relevance to liver fibrosis in BA patients remain to be elucidated further.

Despite the significant results presented herein, we should be aware of some inherent limitations. The most important drawback is the fact that this study is cross-sectional in its design. In this context, it is difficult to determine the underlying mechanisms regulating the causal relationships between increased circulating COMP levels and unfavorable outcome of post-operative BA patients. It is recommended that multi-center prospective cohorts are needed to verify any relationships. Another caveat is the lack of data on co-morbidities of BA that makes it difficult to interpret our result about whether high circulating COMP levels were independently associated with reduced survival of post-operative BA patients with unfavorable outcome. Likewise, given that immunofluorescence staining was restricted, it is difficult to ensure whether COMP protein is expressed in macrophages or other non-parenchymal cells. Corresponding to that point, quantification of COMP protein expression in BA livers with or without fibrotic scarring were both unachievable. To overcome those challenges, Western blot and immunofluorescence staining need to be performed. Furthermore, since the study participants were from hospital-based participants rather than the general population, there might be some risk of selection bias if they had any differences in terms of the studied exposures. Unfortunately, due to ethical issues, direct comparisons between COMP protein expression in the liver of healthy controls and that of BA patients were unachievable as a result of being unable to collect the liver specimens from healthy volunteers. In future studies, this may be overcome by the use of non-BA patients with neonatal cholestasis as controls.

To sum up, this is the first study to provide novel evidence of increased circulating COMP levels in post-operative BA patients—particularly in those with fibrosis. Indeed, circulating COMP levels were found to be independently associated with degree of liver fibrosis in the patients. Interestingly, high circulating COMP levels were observed to be significantly correlated with declined survival of post-operative BA patients with unfavorable outcome. Besides an increment in COMP levels in the circulation, subsequent analysis demonstrated up-regulation of COMP mRNA and protein expressions in BA livers, predominantly in the fibrotic area. Taken together, circulating COMP appears to have potential as a non-invasive biomarker for BA progression—particularly hepatic fibrosis. As currently available data are derived from in-depth analyses of these relationships, future validation with prospective studies is needed to confirm the potential of circulating COMP as a biomarker for progression of liver fibrosis in post-operative BA patients.
